# Ultrasound Guidance for Radial Artery Catheterization: An Updated Meta-Analysis of Randomized Controlled Trials

**DOI:** 10.1371/journal.pone.0111527

**Published:** 2014-11-06

**Authors:** Lu Tang, Fei Wang, Yuxiang Li, Liang Zhao, Huijun Xi, Zhihong Guo, Xiuyun Li, Chengjie Gao, Jian Wang, Lingjun Zhou

**Affiliations:** 1 Department of Anesthesiology, General Hospital of Jinan Military Command, Jinan 250031, China; 2 School of Nursing, Ningxia Medical University, Yinchuan 750004, China; 3 Department of Anesthesiology, the First Affiliated Hospital, Guangxi Medical University, Nanning 530021, China; 4 Digestive Endoscopy Center, Changhai Hospital, Second Military Medical University, Shanghai 200433, China; 5 Department of Nursing, General Hospital of Jinan Military Command, Jinan 250031, China; 6 Institute of Translational Medicine, Second Military Medical University, Shanghai 200433, China; Azienda Ospedaliero-Universitaria Careggi, Italy

## Abstract

**Background:**

Since a previous meta-analysis reported that ultrasound guidance was associated with a higher first-attempt success rate in catheterization of the radial artery, a number of randomized controlled trials (RCTs) have reported inconsistent results. The aim of the present study is to conduct an updated meta-analysis to clarify the role of ultrasound guidance for radial artery catheterization.

**Methods:**

A systematic literature search of PubMed, Embase, and Cochrane Central Register of Controlled Trials was conducted using specific search terms. Eligible studies were RCTs that compared ultrasound guidance with traditional palpation for radial artery catheterization. The Mantel-Haenszel method using the random effects model was adopted in this meta-analysis.

**Results:**

Seven RCTs with 482 patients were included. Compared with traditional palpation, ultrasound guidance significantly increased the first-attempt success rate of radial artery catheterization (RR 1.51, 95% CI 1.07–2.14, *P* = 0.02). Subgroup analyses suggested that the superiority of ultrasound guidance for radial artery catheterization was significant when the technique was operated by experienced users, performed in small children and infants, and on elective procedures in the operating room. In addition, ultrasound guidance significantly reduced mean-attempts to success (WMD −1.13, 95% CI −1.58 to −0.69, *P*<0.00001), mean-time to success (WMD −74.77s, 95% CI −137.89s to −11.64s, *P* = 0.02), and the occurrence of hematoma (RR 0.17, 95% CI 0.07–0.41, *P* = 0.0001).

**Conclusions:**

The present meta-analysis suggests a clear benefit from ultrasound guidance for radial artery catheterization compared with the traditional palpation. Preliminary training and familiarization with the ultrasound-guided technique is needed before applying it for radial artery catheterization, especially for inexperienced operators.

## Introduction

Arterial catheterization is often performed in critically ill patients for continuous hemodynamic monitoring and blood gas sampling in a wide range of locations within the hospital. The radial artery is the preferred site for arterial catheterization due to its superficial course and a low rate of complications [1]. However, insertion of the radial artery catheter with traditional palpation may be technically challenging, often requiring multiple attempts and causing patient discomfort and suffering, particularly in pediatric patients or patients with hypotension, edema, and obesity. Although the procedure is generally safe, complications such as hematoma and infections occur in about 5% cases [2].

Real-time ultrasound guidance can not only visually distinguish arteries, veins, and surrounding structures but predict variant anatomies and assess the patency of a target vessel, and therefore has become an increasingly popular clinical practice [3]. Ultrasound equipment such as the SonoSite 180 plus, the GE Vivid S6 machine and the Flex-Focus 400 anesthesia ultrasonography system was generally used for vascular access. Two different techniques exist for vascular visualization: the long axis in-plane (LA-IP) approach and the short axis out-of-plane (SA-OOP) approach. Recently, a modified version of the SA-OOP approach, termed ‘dynamic needle tip positioning (DNTP)’ was described to be superior to the LA-IP approach in a gelatine phantom [4].

Recently, ultrasound guidance has been recommended for use in cannulation of the internal jugular vein in several national medical agencies and guidelines [5]–[8]. With respect to radial artery catheterization, a previous meta-analysis indicated that the use of ultrasound guidance improved the first-pass success rate [9]. Since then, a number of randomized controlled trials (RCTs) addressing this topic have reported inconsistent results [10]–[12]. In order to provide the latest and more solid evidence and minimize potential bias caused by limited publications, we performed an updated meta-analysis to further investigate the effect of ultrasound guidance for radial artery catheterization *vs.* traditional palpation with respect to first-attempt success and secondary clinical outcomes.

## Methods

The present meta-analysis was performed according to the guidelines of Preferred Reporting Items for Systematic Reviews and Meta-Analyses [13].

### Search strategy and eligibility criteria

Relevant articles in all languages were identified by searching PubMed, Embase, and the Cochrane Central Register of Controlled Trials (April 20, 2014). We used Exploded Medical Subject Headings and the appropriate corresponding keywords “ultrasound”, “ultrasonography”, “ultrasonic” AND “catheterization”, “cannulation”, “catheter”, “catheters”, “insertion” AND “radial artery”. We also checked the reference lists of RCTs and previous meta-analyses identified by the previous searches for additional studies eligible for inclusion.

Two authors independently included RCTs if they compared the real-time 2-D ultrasound guidance technique with the traditional palpation method for radial artery catheterization. RCTs assessing the use of Doppler ultrasonography were excluded. Agreement regarding trial inclusion was assessed using the Cohen К statistic [14].

### Data extraction

Two authors independently extracted the following data from each included trial: first author, year of publication, study design, patient characteristics, operator experience, ultrasound equipment, ultrasound-guided techniques and main outcomes. The ultrasound-guided techniques included the LA-IP approach and the SA-OOP approach. The DNTP technique was classed as the SA-OOP approach [4]. If data needed clarification or were not presented in the publication, the original authors were contacted by E-mail. Extracted data were checked by the third author, and any discrepancy was resolved by discussion.

The primary outcome was the first-attempt success rate. Secondary outcomes included mean-attempts to success, mean-time to success, and the occurrence of hematoma. The definition of each outcome mentioned above was the same as that used in each included trial.

### Risk of bias assessment

The risk of bias of each included trial was assessed using the method recommended by the Cochrane Collaboration [15]. The criteria used for assessment were sequence generation of allocation, allocation concealment, blinding, complete outcome data addressed, no selective outcome reporting and free of other sources of bias.

### Statistical analyses

Differences were expressed as the relative risk (RR) with 95% confidence interval (CI) for dichotomous outcomes, and the weighted mean difference (WMD) with 95% CI for continuous outcomes. The Mantel-Haenszel method with random effects model was used across the pooled analyses. Heterogeneity was estimated using the *I^2^* statistic, and *I^2^*>50% indicated significant heterogeneity [16]. Potential sources of heterogeneity were identified by sensitivity analyses conducted by omitting one study in each turn and investigating the influence of a single study on the overall pooled estimate. Prior subgroup analyses stratified by patients' age, clinical setting, and operator experience were carried out to explore the influence of various clinical factors on the overall pooled estimate. Publication bias was not assessed due to fewer than ten trials included [17]. A two-tailed *P*<0.05 was considered statistically significant, except where otherwise specified. All statistical analyses were performed with RevMan 5.2 (The Nordic Cochrane Centre, Copenhagen, Denmark).

## Results

**Figure 1:**
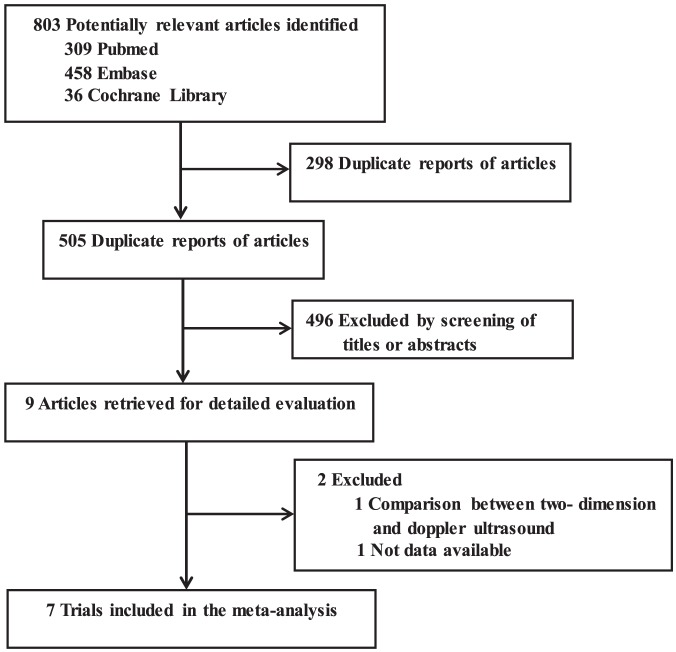
The flow diagram of the selection process of the included studies.

The initial search yielded a total of 803 relevant publications, and the abstracts were obtained for all citations (Figure 1). Finally, seven RCTs with a total of 482 patients fulfilled the criteria to be included in the meta-analysis [10]–[12], [18]–[21]. The Cohen К statistic for agreement on study inclusion was 0.93.

### Study characteristics

Among the seven trials published between 2003 and 2014, four [10], [12], [18], [19] were conducted in Europe, two [20], [21] in North America, and one [11] in Asia. The sample size ranged from 30 to 152. Among the included RCTs, four [10], [12], [18], [20] focused on adult patients and three [11], [19], [21] on small children and infants. Ultrasound guidance was used by experienced operators in four RCTs [10], [12], [19], [21] and in three trials the technique was operated by inexperienced operators [11], [18], [20]. Five RCTs [11], [12], [18], [19], [21] were conducted in the operation theater and two [10], [20] in the emergency ward. The ultrasound-guided LA-IP approach was adopted in one RCT [20] and the SA-OOP approach was used in five RCTs [11], [12], [18], [19], [21]. Details of the included trials are presented in Table 1.

**Table 1:** Characteristics of the included studies.

Study/Year	Country	Population	Patients	Age (y)	Clinical Setting	Operator Experience	Ultrasound Equipment	Ultrasound-guided Approach
Levin et al, 2003 [18]	Israel	Adults	69	UG: 59.9±14.8 Palpation: 66.4±14.3	Elective abdominal, cardiothoracic, vascular surgery and neurosurgery in operating room	Anesthetists with experience of ultrasound-guided central venous catheterization but no experience of ultrasound-guided arterial catheterization	4-MHz transducer of the portable ultrasound device (Site Rite II, Dymax Corporation, Pittsburgh, PA)	short axis out-of-plane
Schwemmer et al, 2006 [19]	Germany	Small children and infants	30	UG: 3.4±2.9 Palpation: 3.3±2.7	Elective major neurosurgery in operating room	Anesthetists with experience of >20 ultrasound-guided arterial catheterization	15-MHz transducer of small parts imaging capability (Sonos 5000; Hewlett-Packard, Andover, MA, USA)	short axis out-of-plane
Shiver et al, 2006 [20]	USA	Adults	60	≥18	Emergency department	Attending physicians with experience of ultrasound-guided peripheral and central venous catheterization but no experience of ultrasound-guided arterial catheterization	SonoSite Ilook 25 (Bothell, WA) US machine with a 5-10 MHz transducer	long axis in-plane
Ganesh et al, 2009 [21]	USA	Children	152	UG: 8.3±5.8 Palpation: 8.3±6.0	Elective abdominal, craniofacial, orthopedic, thoracic surgery and neurosurgery in operating room	Anesthetists with experience of <10 ultrasound-guided arterial catheterization	5–10 MHz transducer of the portable US device (SonoSite 180 plus, SonoSite, Bothell, WA)	short axis out-of-plane
Bobbia et al, 2013 [10]	France	Adults	72	UG: 69 (56–82)† Palpation: 71 (61–85)†	Emergency department	Physicians receiving 3 hours of simulator training on ultrasound-guided arterial puncture	10-MHz transducer of GE Vivid S6 machine (General Electric Company, Fairfield, CT)	Not reported
Ishii et al, 2013 [11]	Japan	Small children and infants	59	1.5 (0.6–2.3)†	Elective cardiac surgery for congenital heart disease in operating room	Anesthetists with experience of ultrasound-guided central venous catheterization but no experience of ultrasound-guided arterial catheterization	2–7 MHz transducer of SonoSite 180 ultrasound imaging device (SonoSite, Bothell, WA)	short axis out-of-plane
Hansen et al, 2014 [12]	Denmark	Adults	40	65.8±16.1	Elective cardiac surgery in operating room	Anesthetists with 20-year experience in transesophageal and transthoracic ultrasonography, 1-year experience with ultrasonography dynamic needle tip positioning	18-MHz transducer of a Flex-Focus 400 anesthesia ultrasonography system (BKMedical, Herlev, Denmark)	short axis out-of-plane

UG  =  ultrasound guidance.

† Median (interquartile range).

### Risk of bias assessment

Randomized sequence was adequately generated in five trials [10], [12], [19]–[21] and was judged to be unclear in two trials [11], [18] due to insufficient information reported. Allocation sequence concealment was adequately conducted in one trial [20] through a sealed envelope, and was identified to be unclear in six trials based on the original publications [10]–[12], [18], [19], [21]. Because of the nature of the intervention, operators could not be blinded to the randomization arm. It was clearly stated that blinded fashion for patients and outcome assessor was conducted in one trial [12]. The numbers and reasons for withdrawal/dropout were reported in detail in all but one trial [10]. None was terminated earlier due to the data-dependent process or other problems, so free of other sources of bias were defined across trials. An overview of the risk of bias assessment is presented in Table 2.

**Table 2:** Assessing risk of bias.

Study/Year	Sequence Generation	Allocation Concealment	Blinding	Complete Outcome Data Addressed	No selective Outcome Reporting	Free of Other Bias
Levin et al, 2003 [18]	Unclear	Unclear	No	Yes	Yes	Yes
Schwemmer et al, 2006 [19]	Yes	Unclear	No	Yes	Yes	Yes
Shiver et al, 2006 [20]	Yes	Yes	No	Yes	Yes	Yes
Ganesh et al, 2009 [21]	Yes	Unclear	No	Yes	Yes	Yes
Bobbia et al, 2013 [10]	Yes	Unclear	No	No	Yes	Yes
Ishii et al, 2013 [11]	Unclear	Unclear	No	Yes	Yes	Yes
Hansen et al, 2014 [12]	Yes	Unclear	Yes	Yes	Yes	Yes

### Primary outcome: first-attempt success

**Figure 2:**
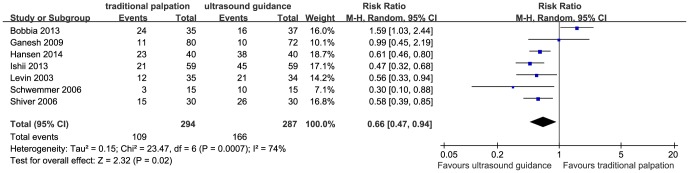
The forest plot depicting first-attempt success.

Data on the outcome were available from all seven trials [10]–[12], [18]–[21]. Compared with traditional palpation, ultrasound guidance significantly increased the first-attempt success rate of radial artery catheterization (RR 1.51, 95% CI 1.07–2.14, *P* = 0.02, Figure 2) with significant heterogeneity (*P* = 0.0007, *I^2^* = 74%). Sensitivity analyses for heterogeneity identified the trial by Bobbia et al [10] as having outlying results. Exclusion of this trial resolved the heterogeneity and the superiority of ultrasound guidance was maintained (RR 1.77, 95% CI 1.49–2.11, *P*<0.00001; *P* for heterogeneity  = 0.48, *I^2^* = 0%).

Subgroup analyses were conducted to investigate the influence of patients' age, clinical setting, and operator experience on the first-attempt success rate. The superiority of ultrasound guidance for radial artery catheterization was significantly evident in the pediatric patients (RR 1.88, 95% CI 1.07–3.31, *I^2^* = 48%) [11], [19], [21], but not in adult patients (RR 1.35, 95% CI 0.85–2.14, *I^2^* = 82%) [10], [12], [18], [20]; in the operating room for elective surgery (RR 1.79, 95% CI 1.44–2.23, *I^2^* = 11%) [11], [12], [18], [19], [21], but not in the emergency ward (RR 1.05, 95% CI 0.39–2.85, *I^2^* = 92%) [10], [20]. In addition, ultrasound guidance was associated with a higher first-attempt success rate when performed by an experienced (RR 1.98, 95% CI 1.04–3.77, *I^2^* = 44%) [12], [19], [21] operator *vs.* an inexperienced operator (RR 1.36, 95% CI 0.84–2.20, *I^2^* = 81%) [11], [18], [20].

### Secondary outcomes

**Figure 3:**
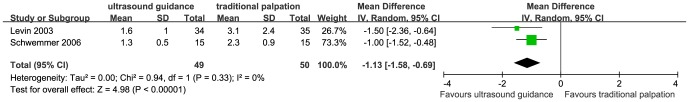
The forest plot depicting mean-attempts to success.

**Figure 4:**
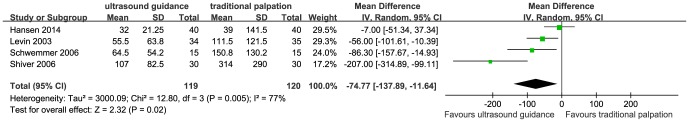
The forest plot depicting mean-time to success.

**Figure 5:**
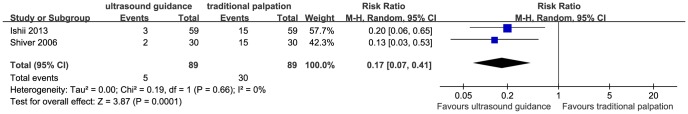
The forest plot depicting the occurrence of hematoma.

Compared with traditional palpation, ultrasound guidance significantly reduced mean-attempts to success (WMD −1.13, 95% CI −1.58 to −0.69, *P*<0.00001, Figure 3) [18], [19], mean-time to success (WMD −74.77s, 95% CI −137.89s to −11.64s, *P* = 0.02, Figure 4) [12], [18]–[20], and the occurrence of hematoma (RR 0.17, 95% CI 0.07–0.41, *P* = 0.0001, Figure 5) [11], [20] in radial artery catheterization. Subgroup analysis on second outcomes was not performed due to limited data available for these outcomes in the included trials.

## Discussion

The present meta-analysis suggests a clear benefit from ultrasound guidance for radial artery catheterization compared with traditional palpation, as manifested by a higher first-attempt success rate, fewer mean-attempts to success, shorter mean-time to success, and reduced occurrence of hematoma. One explanation for the superiority of ultrasound guidance *vs.* traditional palpation is that ultrasonography clarifies the relative position of the needle, the radial artery and its surrounding structures, especially in hypotensive or obese patients. Additionally, the real-time image offered by ultrasonography allows the operator to predict variant anatomies. It is reported that variants in the origin or course of the radial artery are as high as 30% in individuals, although less aberration occur at the distal forearm [22].

Cannulation of the radial artery in infants and small children can be technically challenging, even for experienced operators, especially after repeated unsuccessful attempts causing complications such as hemorrhage and hematoma formation. Among the included trials, three [11], [19], [21] evaluated the effect of ultrasound guidance for radial artery catheterization in infants and small children. One possible reason for the failure to demonstrate the superiority of ultrasound guidance in the study of Ganesh et al [21] is the operator's inexperience, where the rate of successful cannulation at first attempt with the ultrasound-guided technique was only 13.9%, which is significantly lower than 66.7% reported by Schwemmer et al [19] and 76.3% reported by Ishii et al [11]. Although the results of these trials are not consistent, the pooled analysis in our meta-analysis suggests a significantly higher first-attempt success rate under ultrasound guidance (RR 1.88, 95% CI 1.07–3.31) [11], [19], [21], which was not detected in adult patients (RR 1.35, 95% CI 0.85–2.14) [10], [12], [18], [20].

Technically, the operator's experience plays an important role in using ultrasound guidance for radial arterial catheterization. Pooled analyses in the present study suggested that ultrasound guidance significantly increased the first-attempt success rate when performed by an experienced operator (RR 1.98, 95% CI 1.04–3.77) [12], [19], [21] vs. an inexperienced operator (RR 1.36, 95% CI 0.84–2.20) [11], [18], [20], suggesting that there is a need for preliminary training and familiarization with the ultrasound technique before applying it for radial artery catheterization.

Generally, the training of ultrasound technique for vascular access conform to a steep learning curve, and the rate at which competency and proficiency are gained increases rapidly with training and experience [23]. The recently published guidelines recommended by the American Society of Echocardiography and the Society of Cardiovascular Anesthesiologists suggest a minimum of 10 procedures performed under the supervision of an experienced practitioner [7]. The training required may vary between the different indications and significantly more experience is required for pediatric vascular access as compared with adults.

In the process of exploring the potential source of significant heterogeneity, Bobbia et al's study [10] was identified as having outlying results, because the aim of the study was to evaluate ultrasound-guided radial artery puncture for blood gas sampling rather than ultrasound guidance for radial artery catheterization. In some circumstances, the catheter could not be passed successfully into the artery, despite apparent good blood return indicating successful puncture, making any further attempts more difficult. Exclusion of Bobbia et al's study [10] resolved the heterogeneity and the superiority of ultrasound guidance was maintained (RR 1.77, 95% CI 1.49–2.11, *P*<0.00001; *P* for heterogeneity  = 0.48, *I^2^* = 0%).

At present, the LA-IP approach and SA-OOP approach are two main techniques for ultrasound-guided vascular access. The LA-IP approach visualizes the vessel and needle/catheter in the long axis and therefore allows for better visualization of the needle shaft and tip throughout needle advancement, which renders it a theoretically more precise method. The SA-OOP approach images the vessel in its short axis. The main advantage of the SA-OOP approach is that relevant structures and their relationship to each other can be visualized simultaneously side by side during cannulation. The most recently described technique is termed DNTP [4], a modified version of the SA-OOP approach where the transducer and needle are alternately moved so that the needle tip is successively visible or invisible on the screen. The DNTP technique has been shown to require limited training by novices in order to improve precision of vascular access [4]. Among the included trials, one [20] used the LA-IP approach and five [11], [12], [18], [19], [21] used the SA-OOP approach. Of note, the DNTP technique was used in the recently published trial [21]. A recent randomized trial compared the ultrasound-guided LA-IP and SA-OOP approaches for radial artery cannulation, finding that the former significantly increased the first-attempt success rate and shortened the cannulation time [24], which is contrary to ultrasound guidance for central vein catheterization [25]. More RCTs are needed to make a more definite conclusion on the effect of the two ultrasound guidance approaches on radial artery catheterization.

For the purpose of radial artery catheterization, small linear array probes with high-frequency transducers typically are preferred to allow for high-resolution imaging in the near field [23]. The depth on the US machine should be adjusted so the radial artery and the adjacent relevant structures clearly are visualized and identifiable. Gain control should be adjusted so that fine anatomic details can be differentiated. Future advances in imaging technology are needed to invent smaller, higher-frequency probes allowing for higher-resolution imaging and cheaper and better machines.

Differences between the current meta-analysis and the previous meta-analysis [9] should be noted. The previous meta-analysis by Shiloh et al [9] included four RCTs [18]–[21], and our meta-analysis included three more RCTs published since 2011 [10]–[12], which increases the power to detect the true effect of ultrasound guidance for radial artery catheterization. With respect to the risk of bias assessment, the meta-analysis performed by Shiloh et al [9] used the Jadad scale, which is explicitly discouraged currently. Our meta-analysis adopted the method recommended by the Cochrane Collaboration [15]. In addition, second outcomes including mean-attempts to success, mean-time to success and the occurrence of hematoma were evaluated in the present meta-analysis. Besides, subgroup analyses suggested that the benefit of ultrasound guidance for radial artery catheterization was significant when the technique was operated by experienced users not by inexperienced users, performed in small children and infants not in adults, and in elective procedures not under emergency conditions.

At the end of preparing our manuscript, a meta-analysis by Gu et al [26] was published, suggesting that ultrasound guidance is an effective and safe technique for radial artery catheterization. However, several differences should be highlighted here. First, our meta-analysis specifically focused on ultrasound guidance *vs.* traditional palpation for radial artery catheterization and thus Ueda et al's study [27] comparing ultrasound guidance with Doppler-assisted technique was excluded, which was included in Gu et al's meta-analysis [26]. In addition, our meta-analysis included the most recently published RCT by Hansen et al in 2014 [12], which was not included in Gu et al's [26]. Second, in our meta-analysis, subgroup analysis suggested that the benefit of ultrasound guidance for radial artery catheterization was significant when the technique was operated by experienced users not by inexperienced users, which was not reported by Gu et al's [26].

There are several limitations in the present meta-analysis. First, the risk of bias of the included trials is an important issue due to insufficient information reported. Second, there is considerable heterogeneity between the included trials with respect to population characteristics, operators' experience, ultrasound equipment and outcome definition. Third, data on secondary outcomes were limited in the included trials, and thus caution should be taken when interpreting the results. Finally, the geographic regions covered included North America, Europe, and Asia. Therefore, it should be prudent to apply the present results to other regions such as Africa and Latin America.

In conclusion, the present meta-analysis suggests that ultrasound guidance for radial artery catheterization can significantly increase first-attempt success and reduce mean-attempts to success, mean-time to success, and the occurrence of hematoma. Preliminary training and familiarization with the ultrasound-guided technique is needed before applying it for radial artery catheterization, especially for inexperienced operators.

## Supporting Information

Checklist S1 **PRISMA checklist.**
(DOC)
